# Supplementary material for: Pectin Induced Colony Expansion of Soil-Derived Flavobacterium Strains
Source: Front Microbiol. 2021 Apr 6;12:651891. doi: 10.3389/fmicb.2021.651891 (PMC8056085; doi:10.3389/fmicb.2021.651891)
Supplement: Supplementary file 1 [file Data_Sheet_1.PDF]

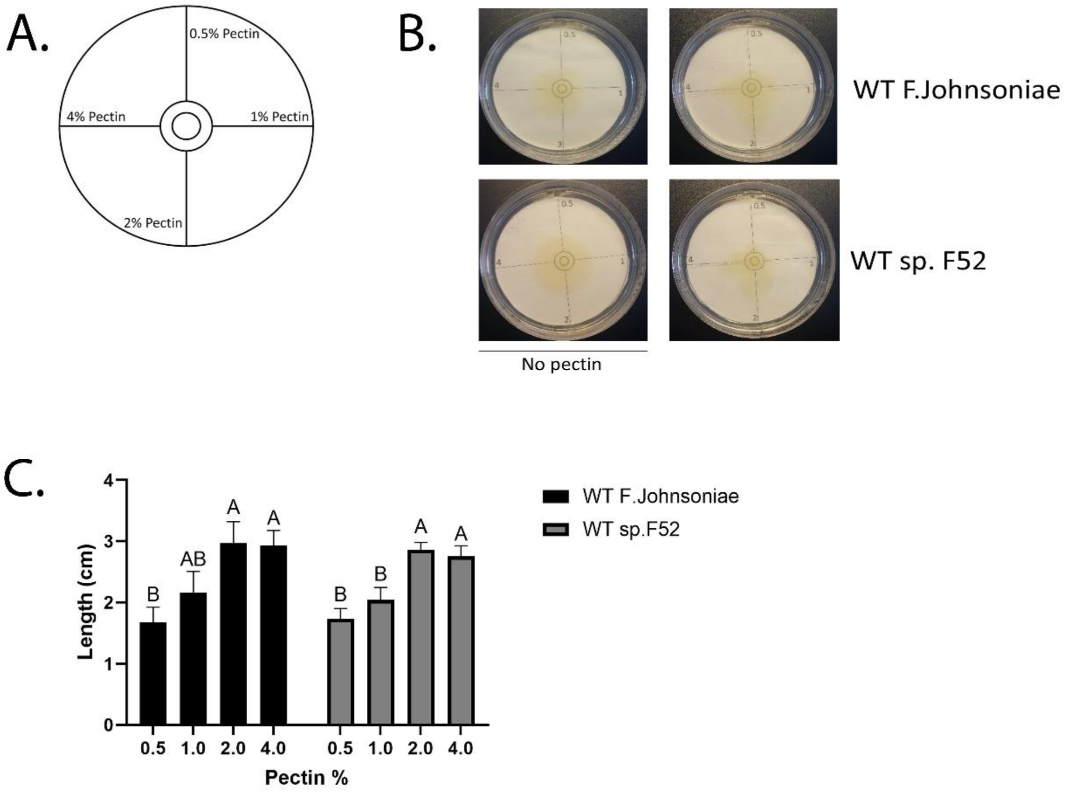

FigS1– Effect of pectin concentration on Flavobacteria colony expansion. (A) Schematic representation of PY2 agar plates streaked with 0.5, 1, 2, 4% pectin as indicated. (B) Wild type (WT) or gliding mutant (ΔgldJ) of *F. johnsoniae*, sp. *F52*, flavobacteria strains were inoculated in the center of PY2 plates streaked with lines of various pectin concentrations (as indicated in A.) or PY2 agar plates streaked with DDW as a control. Bacteria were grown for 48hr at $30^{O}C$ and the distance of the bacterial colony from the center outwards was measured using Fiji. $p<0.05$ by Tukey HSD. N=4. (C) Distance of WT *F. johnsoniae*, *Flavobacterium* sp. F52 strains from the plate center outwards measured on PY2 agar covered with the indicated concentration of pectin. Presented bars show mean and SE values. $p<0.05$ by Tukey HSD.

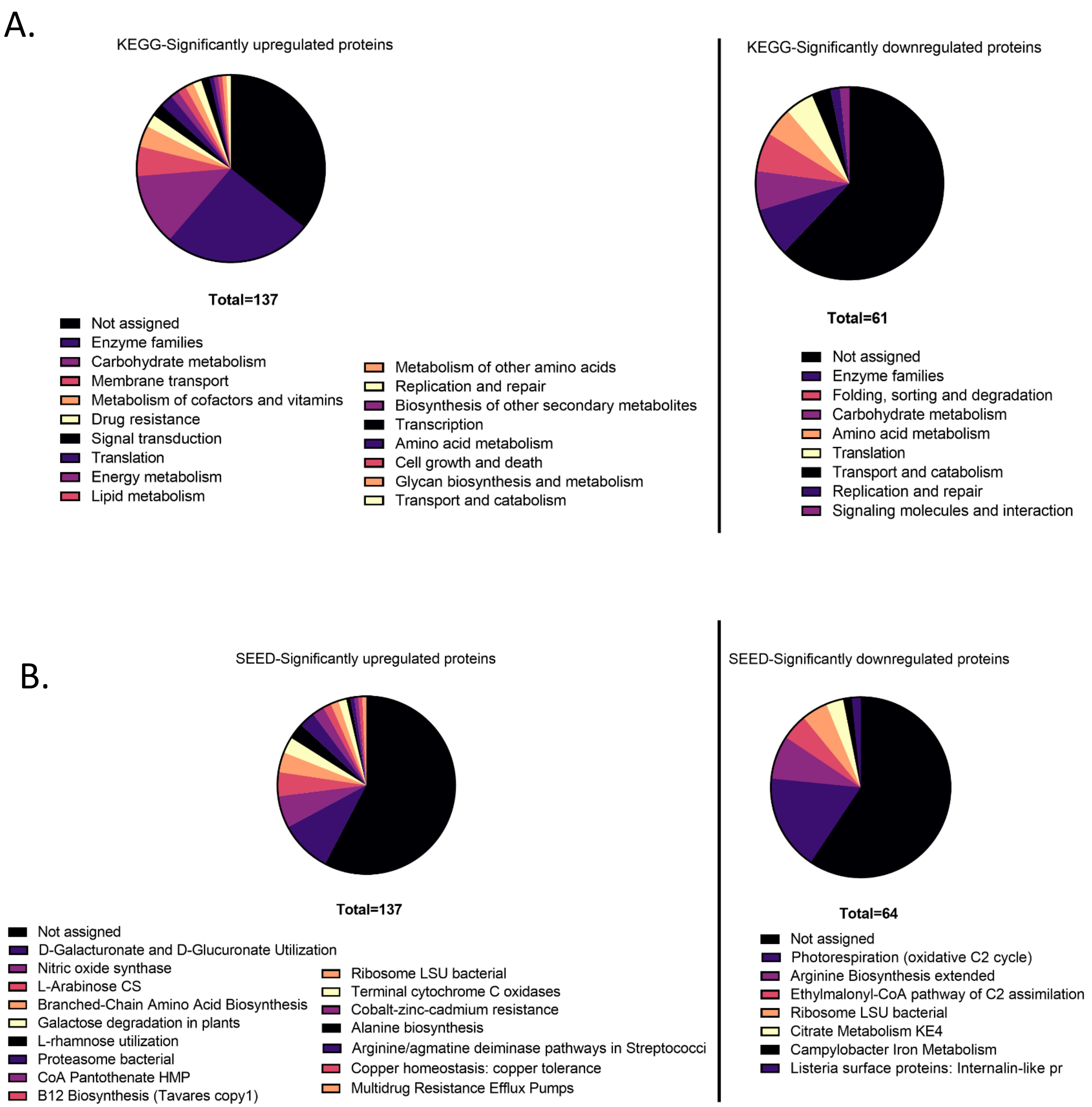
A.
KEGG-Significantly upregulated proteins
Total=137
Not assigned
Enzyme families
Carbohydrate metabolism
Membrane transport
Metabolism of cofactors and vitamins
Drug resistance
Signal transduction
Translation
Energy metabolism
Lipid metabolism
Metabolism of other amino acids
Replication and repair
Biosynthesis of other secondary metabolites
Transcription
Amino acid metabolism
Cell growth and death
Glycan biosynthesis and metabolism
Transport and catabolism
KEGG-Significantly downregulated proteins
Total=61
Not assigned
Enzyme families
Folding, sorting and degradation
Carbohydrate metabolism
Amino acid metabolism
Translation
Transport and catabolism
Replication and repair
Signaling molecules and interaction
B.
SEED-Significantly upregulated proteins
Total=137
Not assigned
D-Galacturonate and D-Glucuronate Utilization
Nitric oxide synthase
L-Arabinose CS
Branched-Chain Amino Acid Biosynthesis
Galactose degradation in plants
L-rhamnose utilization
Proteasome bacterial
CoA Pantothenate HMP
B12 Biosynthesis (Tavares copy1)
Ribosome LSU bacterial
Terminal cytochrome C oxidases
Cobalt-zinc-cadmium resistance
Alanine biosynthesis
Arginine/agmatine deiminase pathways in Streptococci
Copper homeostasis: copper tolerance
Multidrug Resistance Efflux Pumps
SEED-Significantly downregulated proteins
Total=64
Not assigned
Photorespiration (oxidative C2 cycle)
Arginine Biosynthesis extended
Ethylmalonyl-CoA pathway of C2 assimilation
Ribosome LSU bacterial
Citrate Metabolism KE4
Campylobacter Iron Metabolism
Listeria surface proteins: Internalin-like pr

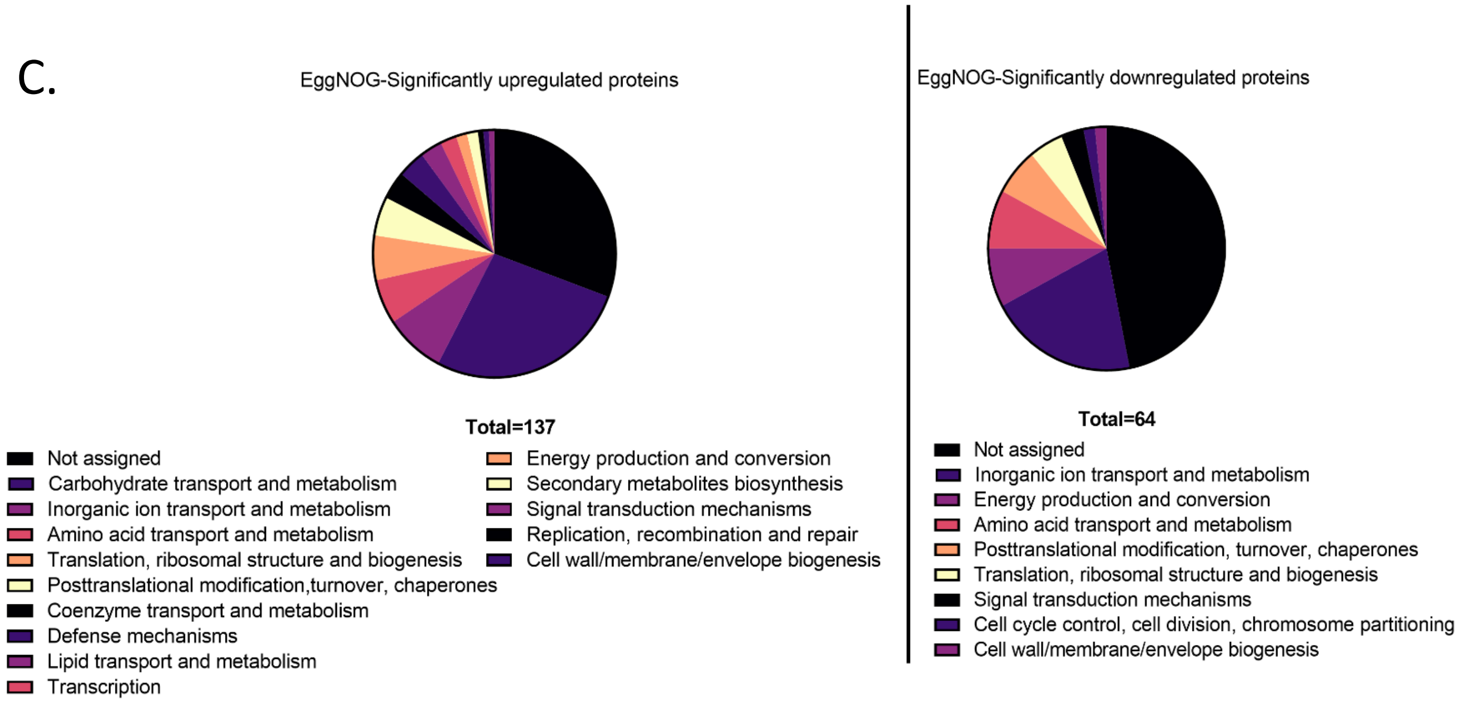

FigS2– Annotation of proteins upregulated and down regulated in response to pectin in *F. johnsoniae*

Proteins that were significantly altered and had fold change higher then 2 (left panel) or lower then 0.5 (right panel) between flavobacteria grown on PY2 with pectin or DDW were annotated using either: (A) KEGG (B) Seed (C) EggNog.

Supplementary Materials and Methods

Functional annotation of significantly induced and repressed proteins

Proteins showing at least two-fold increase or decrease in PY2 media amended with pectin relative to the PY2 media amended with DDW (control) were screened against the non-redundant (nr) NCBI protein database using DIAMOND v0.9.24.125 (Buchfink et al., 2015) to assign functional annotations. Results were then uploaded to MEGAN Ultimate edition software v6.15.2 (Buchfink et al., 2014). For functional annotation, the Kyoto Encyclopedia of Genes and Genomes (KEGG), SEED (Overbeek et al., 2014), and EggNOG (Based on Clusters of Orthologous groups – COG extension) databases were used.

Operon mapping of TonB-associated gene cluster

A TonB gene cluster that was significantly upregulated in the pectin samples (peg4455-4469 by RAST, WP_044048002.1-OXE95634.1 by NCBI annotation, bases 5125269-5130351 in *F. johnsoniae* UW101, complete genome Sequence ID: CP000685.1) was screened for the presence of operons using two independent tools: Operon mapper (Taboada et al., 2018), and FGENESB Suite of Bacterial Operon-Softberry Inc. (Tyson et al., 2004).

Table S1A- Significantly upregulated proteins of *F. johnsoniae* grown on PY2 covered with pectin vs. DDW

NCBI annotaion	gene_number (RAST annoation)	Pectin/DDW: P-Value	Pectin/DDW: Fold change	DNA seq	RAST functional annotation
WP_012024292.1	2323	0.00669	**585.7**	atgaaaaaatcaattctaattctattcattttaaccagctttt	hypothetical protein
WP_012022292.1	192	0.00148	**203.9**	atgaaagcaaaatatatatttagaggactaatagccgta	hypothetical protein
WP_012023625.1	1618	0.01686	**128.9**	atgttgggattaaaattagctacagaccctcgctgggtta	tRNA-(ms[2]io[6]A)-hydroxylase (EC 1.-.-.-)
WP_012024113.1	2127	0.04239	**84.3**	atgttacaactaaaaatcaaaaaaacagccattatttttg	Beta-glucosidase (EC 3.2.1.21)
WP_012025397.1	3584	0.00002	**38.6**	atgaagaatgtaactatcaaattgttagcagctcttgtatt	Alkaline phosphatase (EC 3.1.3.1)
WP_012023204.1	1170	0.00388	**29.9**	atgaaaaattatgtcataggattagactacggaacaga	Ribulokinase (EC 2.7.1.16)
WP_012023111.1	1074	0.00438	**26.7**	atgtcaattaaatcgatagcagcaaagatatttgcccgc	putative auxin-regulated protein
WP_012023206.1	1172	0.00564	**25.8**	atgatagatatatctcaaaaagaagtatggtttgtagtag	L-arabinose isomerase (EC 5.3.1.4)
WP_012024544.1	2583	0.00180	**23.1**	atgaaactaaattttctaaaatcaattgcactgcttggtatt	SclB protein
WP_012022294.1	194	0.00139	**22.1**	atgaattttaaagatttattaaacaaaggagcaaatcgtt	TonB family protein / TonB-dependent receptor
WP_012022711.1	646	0.04622	**19.3**	atgaaaacaataaataaacttatacttattcttctttctctaa	hypothetical protein
WP_012022637.1	570	0.00997	**14.4**	atgaaaaaatcaattttaaatttcgttacagttgttgctttat	FIG00649626: hypothetical protein
WP_012025446.1	3634	0.02934	**12.9**	atgatttttctttaattataccagtgtataatcgtccagatga	Glycosyltransferase
WP_012023596.1	1589	0.00918	**12.8**	atgaaaaaaatgctacttattcttgcattgtcagtttttggct	FIG00657157: hypothetical protein
WP_012022876.1	819	0.00389	**12.1**	atgctagacgaaaccccaaaacgatttgatagaattgtt	transcriptional regulator, putative
SHH12854.1	4459	0.00449	**11.9**	atgttaggaatgattgttacaagcttaagttcttgcagcaa	RagB/SusD domain protein
WP_012023313.1	1287	0.00123	**11.4**	atgaaaaaaaacctacttttctgactatgcttctaattgct	Endo-1,4-beta-xylanase A precursor (EC 3.2.1.8)
WP_012026225.1	4456	0.01930	**11.2**	atgataaagaaaagccattttagttttgcccaaatcatgc	Rhamnogalacturonides degradation protein RhiN
WP_012022957.1	905	0.01684	**10.1**	atgagaacgtgtcttgaatgctctgaaaaacttgtaggca	FIG00930353: hypothetical protein
WP_012026229.1	4460	0.00035	**8.4**	atgaacattcaaaaatcattaaagaaaaaaataaaata	TonB family protein / TonB-dependent receptor
WP_012022293.1	193	0.00954	**8.4**	atgaaacataaattaataatagcaggattgataatttcaa	putative outer membrane protein, probably involved in nutrient binding
WP_012026233.1	4464	0.00003	**7.7**	atggaaaaaattaacagatcaaattcagagttttcaaac	Altronate oxidoreductase (EC 1.1.1.58)
WP_012026234.1	4465	0.00005	**6.8**	atggcaaaatattcaagaatagaggttgcgcagacaa	4-hydroxy-2-oxoglutarate aldolase (EC 4.1.3.16) @ 2-dehydro-3-deoxyphosphogluconate aldolase (EC 4.1.2.14)
WP_012026236.1	4467	0.00006	**6.4**	atgagcgcaaatacattcatacacgacaatttttattaga	Uronate isomerase (EC 5.3.1.12)
WP_012022307.1	209	0.03901	**6.1**	atgtcagatacaatcgaaaaaattaaatgccttattatag	Thioredoxin reductase (EC 1.8.1.9)
WP_012026223.1	4454	0.00058	**6.0**	atgaaaaatataaaaatcgttattcttctcctttccgtttttc	Beta-xylosidase (EC 3.2.1.37)
WP_011921584.1	72	0.03454	**5.9**	atggtaaaatttggatatacaattttgtacgttgaagacgt	related lyases
WP_012026232.1	4463	0.00037	**5.7**	atggcagcgcagaaaaaattgataaaagttcacccaa	Altronate hydrolase (EC 4.2.1.7)
SHL59770.1	2398	0.02051	**5.5**	atgtcacaaaataatatcattttaacaacaggaacatat	Regulator of nucleoside diphosphate kinase
WP_012026691.1	4957	0.01793	**5.5**	atggaaaaatggaaacaaaatttaatcgccgcaattct	TonB-dependent receptor, putative
WP_089477413.1	4508	0.04171	**5.2**	atgaagaaaatagcatgtattgcgcttttagctttactattt	hypothetical protein
OXE95634.1	4469	0.00400	**4.8**	atgacaaaatatagttcaagatacgcgtcaagtccaga	4-deoxy-L-threo-5-hexosulose-uronate ketol-isomerase (EC 5.3.1.17)
WP_012026094.1	4323	0.04956	**4.7**	atgaaattaatacgttttggagaaatcggaaaagaaaa	5-oxopent-3-ene-1,2,5-tricarboxylate decarboxylase (EC 4.1.1.68)
WP_012026239.1	4470	0.00163	**4.6**	atgagtcaaaccgatgctgttacagtaaatcaaaccgtt	Hexuronate transporter
WP_044048002.1	4455	0.00000	**4.6**	atgtcggttttattagtttgcgtaatggctttaacaggctgta	Rhamnogalacturonides degradation protein RhiN
WP_012026071.1	4300	0.00094	**4.4**	atgaaaaagaacatcatcatatcctctttatttctttcaacc	Putative glycosyl hydrolase of unknown function (DUF1680)
WP_012023778.1	1772	0.00746	**4.3**	atgaactacggtaaagaatttaaaaattttgctacaaag	ATP-dependent Clp protease proteolytic subunit (EC 3.4.21.92)
WP_044047683.1	2297	0.01650	**4.1**	atgagagaaaatgacatgttagaaaaagaagagattt	FIG00655294: hypothetical protein
WP_012023203.1	1169	0.00112	**4.0**	atgaaaaaaacactactactattgtttgttacaatttgtata	Alpha-N-arabinofuranosidase 2 (EC 3.2.1.55)
WP_012023735.1	1729	0.00019	**3.8**	atggaaatgacattaacaacaagtgatgagcaggtaa	Alternative cytochrome c oxidase polypeptide CoxO (EC 1.9.3.1); Cytochrome c oxidase, subunit III (EC 1.9.3.1)

WP_091490394.1	1180	0.00210	**3.8**	atggcttttgaattaccacaattaccttatgcatacgatgc	Manganese superoxide dismutase (EC 1.15.1.1)
WP_044048511.1	4466	0.00013	**3.8**	atgagtagagtagttgcatttggagaaatcatgctgcgtt	2-dehydro-3-deoxygluconate kinase (EC 2.7.1.45)
WP_012026231.1	4462	0.00004	**3.7**	atgagcgaaaaagtaaccatttatgatattgccgaaaa	Hexuronate utilization operon transcriptional repressor ExuR
WP_012026237.1	4468	0.01289	**3.5**	atgacaaacttatttgacataaaaggaaaagttgccctt	2-deoxy-D-gluconate 3-dehydrogenase (EC 1.1.1.125)
WP_012024354.1	2387	0.02187	**3.4**	atgttccagaaaattactcttttaacgcttgtattagctgct	hypothetical protein
WP_012026430.1	4683	0.04473	**3.3**	atgacaatgtattcattcgatataaaaaataaaaaccg	Probable Co/Zn/Cd efflux system membrane fusion protein
WP_012024436.1	2473	0.00884	**3.2**	atgaaaatacaagatattcaggcaattcaattattgcttg	oligoribonuclease A, Bacillus type
WP_052295191.1	3036	0.04623	**3.2**	atgaaaaatataaaaataacactttcattattattggtgat	Putative outer membrane protein, probably involved in nutrient binding
WP_044047803.1	3435	0.01612	**3.1**	atgtcaagcaaagaagcatataaaggtgatcttgatac	FIG01101450: hypothetical protein
WP_012026188.1	4419	0.00004	**3.1**	atgataatcggatcaaaccatattgaatctcataacgag	Predicted L-rhamnose isomerase RhaI (EC 5.3.1.14)
WP_012025223.1	3406	0.00852	**3.0**	atgagcattaaaaacaattcatttttcatctctattttatcac	Probable RND efflux membrane fusion protein
WP_012026482.1	4737	0.00920	**2.9**	atgagaatcatttcaggaaaatacaaagggcgccgga	16S rRNA (guanine(966)-N(2))-methyltransferase (EC 2.1.1.171)
WP_012026068.1	4297	0.02085	**2.9**	atgaaaacaaaatttaataaatatatatcactggcacttt	putative outer membrane protein, probably involved in nutrient binding
WP_012026259.1	4490	0.00199	**2.8**	atgaatttaatacgttttgcactccgcaaacccatttccat	RND multidrug efflux transporter; Acriflavin resistance protein
WP_012025845.1	4060	0.01054	**2.7**	atgacagtacatcataatattaccaatggatacctgagta	Endo-1,4-beta-xylanase A precursor (EC 3.2.1.8)
WP_012026180.1	4411	0.04757	**2.6**	atgaaaaatttagttattttattagcagttgcaattcagttaa	Copper homeostasis protein CutF precursor / Lipoprotein NlpE involeved in surface adhesion
SEP16691.1	568	0.04429	**2.6**	ttgctatttatgaaaaaaacacaaactaagattttattagt	Response regulator receiver:Transcriptional regulatory protein, C- terminal
SHF95927.1	1833	0.02412	**2.5**	ttgccaactgaaaatctgaaaaatatgatcatccaaaaa	Methionine aminopeptidase (EC 3.4.11.18)
WP_011921605.1	94	0.00879	**2.5**	atgagccatttagacgatatattaaaatcacgccgcgat	Nicotinate-nucleotide--dimethylbenzimidazole phosphoribosyltransferase (EC 2.4.2.21)
WP_012025858.1	4074	0.00397	**2.5**	atgaaacgatatatttttagaaatttaataataggatctgta	RagB/SusD domain protein
WP_012023590.1	1583	0.00139	**2.4**	atgaaacaagaagaactacaagccatagcctctcaat	Biotin synthesis protein BioC
WP_012022472.1	385	0.04716	**2.4**	atgttgcaattaaatgtaaagaacgaaacatctaggctt	NG,NG-dimethylarginine dimethylaminohydrolase 1 (EC 3.5.3.18)
WP_012026165.1	4396	0.00089	**2.3**	atggaacaaacattaagatggttcggaccaaatgatcc	Mannonate dehydratase (EC 4.2.1.8)
WP_012026655.1	4918	0.00377	**2.3**	atgaatacagcagattttactacaacaatacttgtgagcc	hypothetical protein
WP_012024856.1	2932	0.00615	**2.3**	atgaataccaaaaatagtaccatcagtctagaaacttat	Cysteine desulfurase (EC 2.8.1.7)
WP_012023797.1	1792	0.03633	**2.2**	atgagaaaacttgagaatagcgaattagaaagaaaat	TRNA/rRNA methyltransferase
WP_012022623.1	556	0.01067	**2.2**	atgccattttcagaattatttgataacgaattcaaacaaa	FIG00651957: hypothetical protein
WP_012026675.1	4939	0.02216	**2.2**	atgaaaaaaaatctacttcttaattgcattattctgcaccgtt	hypothetical protein
WP_012023198.1	1163	0.03806	**2.2**	atgaaaaattttgacattaacgaagatccacacagacg	Galactose-1-phosphate uridylyltransferase (EC 2.7.7.10)
WP_012025504.1	3697	0.03226	**2.1**	atgattagaatttcagttttatttttagcttttgctttttttagttgt	hypothetical protein
WP_012022715.1	651	0.00014	**2.1**	atgaaaataaaactatacgccctttctacagttcttacgc	hypothetical protein
WP_012025247.1	3431	0.01325	**2.1**	atgtcgtttttaacatcattaacagtagcaggaaaagatt	hypothetical protein
WP_012024304.1	2335	0.00569	**2.1**	atgatttcatttctggatctaaaaaaaataaatcagccata	Aminotransferase
WP_012026057.1	4285	0.02152	**2.1**	atgggattgaaaattaaaataactgcgatttgttttggaat	putative large secreted protein
WP_012024919.1	3003	0.00072	**2.1**	atggcaaattatttcaacacactaccacttagattacaat	Ketol-acid reductoisomerase (EC 1.1.1.86)
WP_012025852.1	4068	0.00074	**2.1**	atgaaagtactctataacctattagcgatttttttaattcttac	glycoside hydrolase, family 43
WP_044047537.1	1189	0.02710	**2.0**	atggcattaaggcgagttgttgtaacaggattaggtgca	3-oxoacyl-[acyl-carrier-protein] synthase, KASII (EC 2.3.1.41)
WP_012026676.1	4940	0.02970	**2.0**	atgtcaacagcaaaaaaagattataaaagaatcacaa	3-methyl-2-oxobutanoate hydroxymethyltransferase (EC 2.1.2.11)
WP_012022972.1	922	0.03873	**2.0**	atgaaaaaagttatcatcacattattgtttttagtatcatttg	hypothetical protein
WP_012026682.1	4946	0.03967	**2.0**	atgaaattagacatattagcctttggcgcacatccggatg	LMBE-RELATED PROTEIN

WP_012026316.1	4551	0.04159	**2.0**	atgaaaaaaatagttgaataccgcaagttactaaacgt	chaperone with DnaK; heat shock protein
WP_007803649.1	405	0.02033	**2.0**	atggtacaacaagaatcaagactaaaagtagcagata	LSU ribosomal protein L14p (L23e)

Table S1B- Significantly downregulated proteins of *F. johnsoniae* grwon on PY2 covered with pectin vs. DDW

NCBI annotation	gene_number (RAST annoation)	Pectin/DDW: P-Value	Pectin/DDW: Fold change	DNA seq	RAST functional annotation
WP_011921578.1	2219	0.01329	**0.0**	atggattcagaattaaattcgaaagtcaatctgcaggaa	Possible Galanin
WP_012022355.1	1796	0.01684	**0.0**	atgaaaaaagtatttttaagtttagctgttgttgctgttttaac	hypothetical protein
WP_012022481.1	2768	0.00263	**0.1**	atgaaaaatacagaaacaaaaaatactaaagatacg	protein of unknown function DUF892
WP_012022486.1	2790	0.02104	**0.1**	atgaaaacagatacaaagggaaaccagcgtcagga	hypothetical protein
WP_073099567.1	2317	0.00190	**0.1**	atgaaaaagttatattatacagcaaacgtactacttattct	ErfK/YbiS/YcfS/YnhG family protein
WP_012022550.1	3180	0.01144	**0.2**	atggagccacttatttattatccgacttttgagccgccaac	hypothetical protein
WP_012023095.1	3303	0.00112	**0.2**	atgaaaaattttcaggatgaaaagcagcgagatctgct	Catalase (EC 1.11.1.6)
WP_012023270.1	4631	0.01186	**0.2**	atgaatcttattaaaagaaacggaagccatgtgccggc	Small heat shock protein
WP_012022355.1	263	0.00189	**0.2**	atgaaaacaaaattcttaaaactttcgcttttagcactatt	hypothetical protein
WP_044048264.1	1817	0.00385	**0.2**	atgatacatacagatgaaaccacacaagaagctgttaa	hypothetical protein
WP_012023815.1	2422	0.01124	**0.2**	atgactccaaatatcggaatatctcccgccaacttaaaa	Non-specific DNA-binding protein Dps / Iron-binding ferritin-like antioxidant protein / Ferroxidase (EC 1.16.3.1)
WP_012023822.1	3937	0.00564	**0.2**	atgaatcttatcaaaagaaatgcaaaccagcatcgtgc	Small heat shock protein
WP_012023992.1	1243	0.02321	**0.2**	ttgacacagttaaatctgtctaaaaacagagctttaacct	Internalin-like protein (LPXTG motif) Lmo0331 homolog
WP_012024192.1	3531	0.00304	**0.2**	atgggagatcataaagaccttactgaagaatttgctgtca	Pyridoxamine 5'-phosphate oxidase (EC 1.4.3.5)
WP_012024286.1	5085	0.00000	**0.3**	atggaatacaaagtagtaccattcgtagcttcaatcgatc	hypothetical protein
N/A	5088	0.00065	**0.3**	ttgggaggaattgtttttgcaactggagctttggctgggat	hypothetical protein
WP_012024386.1	3238	0.02224	**0.3**	atggaacacgcaacacagcagtattcaaaagaggtac	protein of unknown function DUF305
WP_012024486.1	3304	0.02676	**0.3**	atgaaaaattcagaaaaaccaaataatacaaaagctg	protein of unknown function DUF892
WP_012024493.1	5084	0.04047	**0.3**	atgtttaaaaaagtaataattgctgaagatatcgacgca	hypothetical protein
WP_012024711.1	4786	0.02762	**0.3**	atgaaagtaatattgattgttttgtgtttttttacggctggtttt	hypothetical protein
WP_012024727.1	1809	0.00592	**0.3**	atgaaaaaccaattagaaattaccgaaacggcaatgg	Malate synthase (EC 2.3.3.9)
WP_012025066.1	4223	0.00011	**0.3**	atgaactataacaaacttttactttgtcttttatttacaatgca	hypothetical protein
SHK65708.1	2530	0.00116	**0.3**	atgaaaaataaattactttggtttttagctgttttcactgtttcg	Mechanosensitive ion channel family protein
WP_012025127.1	3541	0.00945	**0.3**	atgaaaaagatactattagccagtaaagcaattttagga	putative exported protein
WP_012025128.1	1810	0.01642	**0.3**	atgaaaacaacagaagacagaattcaggaattgatta	Isocitrate lyase (EC 4.1.3.1)
WP_012025137.1	5248	0.00410	**0.3**	atgagaaatataaaaatagctgcggcagttacattatta	hypothetical protein
WP_012025282.1	407	0.01798	**0.3**	atgaaacaatcagaaataaaagatctttctgcagcgga	LSU ribosomal protein L29p (L35e)
WP_012025337.1	476	0.01318	**0.3**	atgagtttcaccatttcttcagataaaaagaaatcattact	Ferric siderophore transport system, periplasmic binding protein TonB
WP_012025345.1	66	0.03714	**0.3**	atgaaattatacaaaatagcaaccgttcaatatgtaaat	Cell division inhibitor
WP_073409277.1	437	0.03784	**0.4**	atgtctgaaattgcccttatttcagctagaaaaaaccgac	Magnesium and cobalt efflux protein CorC
WP_012025413.1	3314	0.01428	**0.4**	atggcaacaacttacaaaacgcctggagtatatgttgag	Phage tail sheath protein FI
WP_012025418.1	3606	0.01249	**0.4**	atgaaaaaagtagtattagcttatagcggaggattagat	Argininosuccinate synthase (EC 6.3.4.5)
WP_012025595.1	2414	0.01205	**0.4**	atgaaagcatctagcacaattttaggttttgcagcagcgg	Gas vesicle protein
WP_012025730.1	4990	0.00398	**0.4**	atgcataattatttagaaacaaatttaaaaattgaaacta	hypothetical protein
WP_012026000.1	1058	0.01935	**0.4**	atggcatttgaagagttaagagaaaatactgaaaaaat	FIG00654500: hypothetical protein
WP_012026385.1	3793	0.00828	**0.4**	atgagtttaggtgatttatttccagacaatctgaaagaag	hypothetical protein
WP_012026531.1	4860	0.01204	**0.5**	atggaaaacaaacccgaagaaattactcctgagaaa	Low-complexity acidic protein, XCC2875 type
WP_089477404.1	3601	0.02595	**0.5**	atgaactacatctcaataaaagatatcgactcattatcaa	Ornithine carbamoyltransferase (EC 2.1.3.3)
ABQ07757.1	2523	0.00426	**0.5**	atgaaaaaagtactaacactaacggcccttgtttttacgg	hypothetical protein
WP_012026811.1	1992	0.00004	**0.5**	atgaaaaaattatttatcgcagcaatgttatttgttggaata	hypothetical protein
WP_044048134.1	3524	0.02029	**0.5**	atgaatggtaactttaaaagaaatctgcttataagttcatt	Signal transduction histidine kinase
WP_012026814.1	3466	0.00608	**0.5**	atgataaaatacagcccacaaaattactttaaatgcctg	Antifreeze protein
WP_012026963.1	397	0.01510	**0.5**	atggctaaattattagtaaaacaagtaagaagcaagat	LSU ribosomal protein L30p (L7e)

Table S2- Fold change of proteins related to *F. johnsoniae* gliding machenary in pectin vs. DDW

Protein	NCBI annotation	Gene Sequence	# unique peptides	Pectin/DDW: P-Value	Pectin/DDW: Fold change	Annotation by RAST
RemC	WP_012022324.1	atgaaaagaaaaatactttttcttgg	2	0.08363	**13.0**	Glycosyltransferase (EC 2.4.1.-)
Wzc	WP_012022466.1	atgttagatataaaagatttttccatt	19	0.38789	**0.9**	Tyrosine-protein kinase Wzc (EC 2.7.10.2)
Wza	WP_012022467.1	atgacaaaaaatagcttttatatact	5	0.14301	**1.2**	Polysaccharide export outer membrane protein
SprF	WP_012023064.1	atgatgttatctaaaaaaattattac	4	0.06417	**1.2**	FIG00654737: hypothetical protein
SprB	ABL60886.1	atgaaaaaacctactattttaagaa	27	0.00858	**1.3**	internalin, putative
SprD	WP_012023066.1	atgaagaaaattttactattcataa	28	0.06564	**0.6**	TolA protein
SprC	WP_012023067.1	atgattcaaaaaactactttatctttt	7	0.62616	**0.9**	FIG00654018: hypothetical protein
RemG	WP_012023069.1	atgaaaaaaattactcaaatgatct	6	0.19823	**1.1**	FIG00653638: hypothetical protein
RemF	WP_012023070.1	atgaaagggaaaattattttatttag	1	0.25834	**0.7**	FIG00655764: hypothetical protein
GldE	WP_012023105.1	atgaaaatttcgataggaaacgac	7	0.09691	**0.7**	Ribose 5-phosphate isomerase B (EC 5.3.1.6)
SprE	WP_012023137.1	ttgtataatggaggtcttggacttga	13	0.06601	**0.8**	TPR domain protein
SprT	WP_012023545.1	atgagaaaaattgtaatcgtcatttt	4	0.49504	**1.1**	PorT protein
GldA	WP_012023594.1	atgtcgatagaagtaaacagtatat	4	0.54640	**1.1**	ABC transporter, ATP-binding protein
GldD	WP_012023616.1	atgttaaaaaaaatcctttcagtaa	2	0.25781	**1.6**	GldD
PorV	WP_012023631.1	atgaaaaaaaatatcgcttctattaa	15	0.19036	**1.4**	FIG00650346: hypothetical protein
SprA	WP_012023729.1	atggagttagaaaatcctccaagc	28	0.03250	**1.1**	FIG00648977: hypothetical protein
RemB	WP_012023733.1	gtgaataagttatttttatcttttatcat	4	0.10729	**2.1**	FIG00654784: hypothetical protein
GldB	WP_012023869.1	atgaaaatgtatcgctttgtagtggt	6	0.07962	**2.7**	GldB
GldC	WP_012023870.1	atgtcaaatacaataaaatcagaa	1	0.83912	**0.9**	GldC
GldJ	WP_012023929.1	atgaagaagtttattgcatttgcagc	19	0.05145	**0.9**	GldJ
GldL	WP_012023930.1	atggcattattaagtaaaaaagtta	23	0.27486	**1.1**	FIG00648934: hypothetical protein
GldM	WP_012023931.1	atgtatctggttttcatcgcaatgtta	53	0.00280	**1.1**	FIG00649784: hypothetical protein
GldN	WP_012023932.1	atgaaagtaagaaatttttaatagc	15	0.96310	**1.0**	GldN
GldO	WP_012023933.1	atgaaagtaaggaatttttaattgc	15	0.02686	**0.7**	GldN
GldI	WP_012024435.1	atgaactacttaaaaatcagcattt	4	0.47280	**1.0**	GldI
GldG	WP_012024782.1	atgaaagcatctaataaattaaatc	11	0.64396	**1.0**	gliding motility protein GldG
RemI (RemG-paralog)	WP_012025180.1	atgatttgtttactgctgacagctgc	7	0.34787	**1.8**	FIG00653638: hypothetical protein

Table S3- Pectin induced TonB regulated gene clusters

Cluster 1

NCBI annotation	# unique peptides	Pectin/DDW: P-Value	Pectin/DDW: Fold change	Gene Sequence	Signal Peptide?	RAST annotaion
WP_012026221.1	1	0.05681	**18.2**	atgaaatatttacttctattattaatttcctatgtttcatttgctcagcaaaataattttccaagagcaaa	Y	Polygalacturonase (EC 3.2.1.15)
WP_012026223.1	5	0.00058	**6**	atgaaaaatataaaaatcgttattcttctcctttccgtttttcttttcaaaaaaatacggcacaagcc	Y	Beta-xylosidase (EC 3.2.1.37)
WP_044048002.1	11	0	**4.6**	atgtcggttttattagtttgcgtaatggctttaacaggctgtaaagtaacttcacaagaaacggcaa	Y	Rhamnogalacturonides degradation protein RhiN
WP_012026225.1	5	0.0193	**11.2**	atgataaagaaaagccattttagttttgcccaaatcatgcttataagtatgatgctgttttcattgaat	Y	Rhamnogalacturonides degradation protein RhiN
WP_012026226.1	2	0.08763	**7.4**	atgaactataaaaaaacagtctttgcatttttcctgttgtacagcatttttactgtaggtcagaacaa	Y	Rhamnogalacturonides degradation protein RhiN
SHH12854.1	7	0.00449	**11.9**	atgttaggaatgattgttacaagcttaagttcttgcagcaattatatagaggaggaaagtttatcaa	N	RagB/SusD domain protein
WP_012026229.1	28	0.00035	**8.4**	atgaacattcaaaaatcattaaagaaaaaaataaaatacaatcttgtatttttatttttcctgaatttt	Y	TonB family protein / TonB-dependent receptor
WP_012026230.1	2	0.13578	**2.8**	ttgactatatcatgctcaaaaaaaattactcctgaagctgaaactgatccgtggaaaactatgga	N	Polygalacturonase (EC 3.2.1.15)
WP_012026231.1	6	0.00004	**3.7**	atgagcgaaaaagtaaccatttatgatattgccgaaaaactaaatatcactgcagctactgtttc	N	Hexuronate utilization operon transcriptional
WP_012026232.1	12	0.00037	**5.7**	atggcagcgcagaaaaaattgataaaagttcacccaaccgacaatgtagcggttgctttggtg	N	Altronate hydrolase (EC 4.2.1.7)
WP_012026233.1	14	0.00003	**7.7**	atggaaaaaattaacagatcaaattcagagttttcaaacaggcttccaattaaaattgtacaattt	N	Altronate oxidoreductase (EC 1.1.1.58)
WP_012026234.1	3	0.00005	**6.8**	atggcaaaatattcaagaatagaggttgcgcagacaatgaaagataacggaatggtgccgtt	N	4-hydroxy-2-oxoglutarate aldolase (EC 4.1.3.16) @ 2-dehydro-3-
WP_044048511.1	6	0.00013	**3.8**	atgagtagagtagttgcatttggagaaatcatgctgcgtttatcgacagaaagacatttacgttttt	N	2-dehydro-3-deoxygluconate kinase (EC 2.7.1.45)
WP_012026236.1	15	0.00006	**6.4**	atgagcgcaaatacattcatacacgacaatttttattagaaaataaatacgctgaagagttatat	N	Uronate isomerase (EC 5.3.1.12)
WP_012026237.1	11	0.01289	**3.5**	atgacaaacttatttgacataaaaggaaaagttgcccttattacaggaagtacgcacggactgg	N	2-deoxy-D-gluconate 3-dehydrogenase (EC 1.1.1.125)
OXE95634.1	7	0.004	**4.8**	atgacaaaatatagttcaagatacgcgtcaagtccagaagctgtaaaaaaatatgatacacaa	N	4-deoxy-L-threo-5-hexosulose-uronate ketol-isomerase (EC 5.3.1.17)
WP_012026239.1	3	0.00163	**4.6**	atgagtcaaaccgatgctgttacagtaaatcaaaccgttaagtccgcaggaagatatcgttgga	N	Hexuronate transporter
WP_012026240.1	5	0.73526	**1**	ttgaaaaacacaaaaaccctttatttactcactgtcctgctttttcaggaattgtattacattcaagt	Y	Xylanase

Cluster 2

NCBI annotation	# unique peptides	Pectin/DDW: P-Value	Pectin/DDW: Fold change	Gene Sequence	Signal Peptide?	RAST annotaion
WP_012022292.1	10	0.00148	**203.9**	cgtaacagagattttagttcagccaggtaatataacccacaccataacagcagaagaaaaag	Y	hypothetical protein
WP_012022293.1	15	0.00954	**8.4**	ttaaagcatggggtgatgttcctgctcgttttgaacctatcactacagcaactttatatttgcctaaat	Y	putative outer membrane protein, probably involved in nutrient binding
WP_012022294.1	34	0.00139	**22.1**	gtaacatcagcagagggttcgcctgatgccgatatacgaattagagttcgtggaggaggatcat	Y	TonB family protein / TonB-dependent receptor

Cluster 3

NCBI annotation	# unique peptides	Pectin/DDW: P-Value	Pectin/DDW: Fold change	Gene Sequence	Signal Peptide?	RAST annotaion
WP_012022634.1	3	0.10566	**1.8**	atgaaaatcaattttaaaaaaacgtacaaatttgctataaaatcggcattgtatatcagtcttttctc	N	Phosphate regulon sensor protein PhoR (SphS) (EC 2.7.13.3)
SEP16691.1	3	0.04429	**2.6**	ttgctatttatgaaaaaaacacaaactaagattttattagttgacgacgaaccggatatcttagaa	N	Response regulator receiver:Transcriptional regulatory protein, C-terminal
WP_012022636.1	30	0.05111	**3.6**	atgaacaatgatccgttgccttttgcaaatgttttagttaaaggtacaaacattagcgtaaataccg	N	TonB-dependent receptor
WP_012022637.1	22	0.00997	**14.4**	atgaaaaaatcaattttaaatttcgttacagttgttgctttatcaggaggtttacttacaagctgttcta	Y	FIG00649626: hypothetical protein
WP_012022639.1	1	0.00753	**47.1**	ttgattacagccagcgatatatttacaatttcaagccataaacaatttgaaaaaacagcattaaa	N	Long-chain-fatty-acid--luciferin-component ligase (EC 6.2.1.19)

Cluster 4

NCBI annotation	# unique peptides	Pectin/DDW: P-Value	Pectin/DDW: Fold change	Gene Sequence	Signal Peptide?	RAST annotaion
WP_012026068.1	5	0.02085	**2.9**	atgaaaacaaaatttaataaatatatatcactggcacttttacttattgtaggtgcttcgtgcagcga	Y	putative outer membrane protein, probably involved in nutrient binding
WP_012026069.1	5	0.05904	**5.4**	atgaaacaagcacttttaaagagatgtagttttcttttgtttgcactgatgagtgtaattacttatgctc	Y	TonB family protein / TonB-dependent receptor
WP_012026070.1				atgaaatacattctaaccttatttttaataacaacactttcaatatcagcccaaacgcttgataacaaattgacattaa		rhamnogalacturonan acetylesterase

WP_012026071.1	2	0.00094	**4.4**	atgaaaaagaacatcatcatatcctctttatttctttcaaccgtaatctttgcgcagaacaaaggttt	Y	Putative glycosyl hydrolase of unknown function (DUF1680)
WP_012026072.1	7	0.18079	**2.5**	atgataaagaaattaataagcggtgctgttttgtccttgcttttgaccacaaacggacaagcacaa	Y	COG3866 Pectate lyase

*Validated by qPCR

Table S4- Pectin induced up regulated SusC/D proteins in *F. johnsoniae*

SusC-like protein				
**NCBI annotation**	**DNA seq.**	**fold change**	**p-Value**	**Predicted susbtrate**
WP_012022294.1	atgaattttaaagatttattaaacaaaggagcaa	22.12	0.00139	
WP_012026069.1	atgaaacaagcacttttaaagagatgtagttttct	5.38	0.05904	pectins
WP_012026168.1	atgtgtacagaccgaaaaatcaaatcgtttaaa	102.43	0.00484	
WP_012026229.1	atgaacattcaaaaatcattaaagaaaaaaat	8.39	0.00035	pectins

SusD-like protein				
**NCBI annotation**	**DNA seq.**	**fold change**	**p-Value**	**Predicted susbtrate**
WP_012022293.1	atgaaacataaattaataatagcaggattgata	8.36	0.00954	
WP_012024115.1	atgaaatatagttttaaaataaaaacattaggag	11.61	0.03876	
WP_052295191.1	atgaaaaatataaaaataacactttcattattatt	3.22	0.04623	peptides
WP_012025858.1	atgaaacgatatatttttagaaatttaataatagg	2.46	0.00397	hemicelluloses
WP_012026068.1	atgaaaacaaaatttaataaatatatatcactgg	2.87	0.02085	pectins
SHH12854.1	atgttaggaatgattgttacaagcttaagttcttgc	11.86	0.00449	pectins

TableS5- List of primers for qPCR

Primer name	NCBI acession number	RAST annotation	Forward primer	Reverse primer	Tm	Amplicon length
**Target genes**						
TonB 445	WP_012022294.	TonB family protein / TonB-dependent receptor	GGGTATAGACTGCCTCCTGTAA	TCCTCCTCCACGAACTCTAAT	62	92
Transcript_regul YafY fmaily	WP_012022876.	transcriptional regulator, putative	GGGTATAGACTGCCTCCTGTAA	GCGTAGTGTGTTCCCAAAGA	63/62	110
auxin_regulted protein	WP_012023111.	putative auxin-regulated protein	ACAGCGGGTACCAACATTGTAA	CGTGGTACGGCAGTCCATT	58	79
SusD_73	SHH12854.1	RagB/SusD domain protein	CCTGCCGATGCAACCTATAA	AACCACGGATTTCCTCCATAAA	62	98
TonB_260	WP_012026229.	TonB family protein / TonB-dependent receptor	GCTTTCAAACGCAGGAAGTAAG	GAACCGTATCCTACAACCACTAC	62	106
TonB_2144	WP_012026069.	TonB family protein / TonB-dependent receptor	GGCGCTGGCTTGTTCTTTAT	TGATTGTTGCATCCCATTTAATATCT	58/59	132
Pectate-lyase	WP_012026072.	COG3866 Pectate lyase	AACTGACGGAGGAGCAAAC	ACCTTCGTGTTGTGCATTTAAG	62	100
RemA_847	WP_012022896.	internalin, putative RemA	GGAGAGACAAATAGCGGTACAA	ATGGCCTATCCAGGTGTTATTT	62	102
**Refrence genes**						
Electron transfer flavoprotein, alpha subunit- ETF	WP_012023552.	Electron transfer flavoprotein, alpha subunit, Threonine synthase	TTTAACCCGACACTTGGAGAC	CGATATCAGCATCGGCAATAGA	62	94
DNA gyrase subunit B (EC 5.99.1.3) -GyrB	WP_012024321.	DNA gyrase subunit B	GAGAGGTTGTATCTCCGGTTTC	GAGCCTGAGCTGCTAAGATTAC	62	117
16S rRNA		16S rRNA	CGGCAACGAGCGCAACCC	CCATTGTAGCACGTGTGTAGCC	55	130

Table S6 - Predicted operons in TonB related cluster 1

Tool 1-

Operon Mapper: Bacterial Operon Prediction

Operon	NCBI annotation	Sequence	gene function	start	end	Strand	Operon mapper annotation
1	OXE95634.1	atgacaaaa	4-deoxy-L-threo-5-hexosulose-uronate ketol-isomerase (EC 5.3.1.17)	215	1054	+	5-keto 4-deoxyuronate isomerase
1	WP_012026237.1	atgacaaac	2-deoxy-D-gluconate 3-dehydrogenase (EC 1.1.1.125)	1059	1850	+	R] Dehydrogenases with different specificities
1	WP_012026236.1	atgagcgca	Uronate isomerase (EC 5.3.1.12)	1875	3278	+	Glucuronate isomerase
1	WP_044048511.1	atgagtaga	2-dehydro-3-deoxygluconate kinase (EC 2.7.1.45)	3432	4478	+	Sugar kinases, ribokinase family
1	WP_012026234.1	atggcaaaa	4-hydroxy-2-oxoglutarate aldolase (EC 4.1.3.16) @ 2-dehydro-3-deoxy	4491	5159	+	2-keto-3-deoxy-6-phosphogluconate aldolase
1	WP_012026233.1	atggaaaaa	Altronate oxidoreductase (EC 1.1.1.58)	5333	6817	+	Mannitol-1-phosphate/altronate dehydrogenases
1	OXE95634.1	atgacaaaa	4-deoxy-L-threo-5-hexosulose-uronate ketol-isomerase (EC 5.3.1.17)	6856	8478	+	Altronate dehydratase
2	WP_012026231.1	atgagcgaa	Hexuronate utilization operon transcriptional repressor ExuR	8719	9747	-	Transcriptional regulators
3	WP_012026230.1	ttgactatatc	Polygalacturonase (EC 3.2.1.15)	10038	11465	+	Endopolygalacturonase
3	WP_012026229.1	atgaacattc	TonB family protein / TonB-dependent receptor	11511	14615	+	
3	SHH12854.1	atgttaggaa	RagB/SusD domain protein	14628	16301	+	
4	WP_012026227.1	ttgattttaatg	Pectinesterase (EC 3.1.1.11)	16406	17362	+	Hydrolases of the alpha/beta superfamily
4	WP_012026226.1	atgaactata	Rhamnogalacturonides degradation protein RhiN	17383	18603	+	Predicted unsaturated glucuronyl hydrolase
4	WP_012026225.1	atgataaaga	Rhamnogalacturonides degradation protein RhiN	18616	19821	+	Predicted unsaturated glucuronyl hydrolase
4	WP_044048002.1	atgtcggttta	Rhamnogalacturonides degradation protein RhiN	19871	21064	+	Predicted unsaturated glucuronyl hydrolase

Tool 2-

FGENESB: Bacterial Operon and Gene Prediction

Operon	NCBI annotation	Sequence	gene function	start	end	Strand	score
1	OXE95634.1	atgacaaaa	4-deoxy-L-threo-5-hexosulose-uronate ketol-isomerase (EC 5.3.1.17)	215	1054	+	470
1	WP_012026237.1	atgacaaac	2-deoxy-D-gluconate 3-dehydrogenase (EC 1.1.1.125)	1059	1850	+	587
1	WP_012026236.1	atgagcgca	Uronate isomerase (EC 5.3.1.12)	1875	3278	+	725
2	WP_044048511.1	atgagtaga	2-dehydro-3-deoxygluconate kinase (EC 2.7.1.45)	3456	4478	+	687
2	WP_012026234.1	atggcaaaa	4-hydroxy-2-oxoglutarate aldolase (EC 4.1.3.16) @ 2-dehydro-3-deoxy	4491	5159	+	422
3	WP_012026233.1	atggaaaaa	Altronate oxidoreductase (EC 1.1.1.58)	5369	6817	+	1054
3	WP_012026233.1	atggaaaaa	4-deoxy-L-threo-5-hexosulose-uronate ketol-isomerase (EC 5.3.1.17)	6856	8478	+	1196
4	WP_012026231.1	atgagcgaa	Hexuronate utilization operon transcriptional repressor ExuR	8719	9747	-	651
5	WP_012026230.1	ttgactatatc	Polygalacturonase (EC 3.2.1.15)	10038	11465	+	717
5	WP_012026229.1	atgaacattc	TonB family protein / TonB-dependent receptor	11511	14615	+	1667
5	SHH12854.1	atgttaggaa	RagB/SusD domain protein	14646	16301	+	1049
6	WP_012026227.1	ttgattttaatg	Pectinesterase (EC 3.1.1.11)	16406	17362	+	335
6	WP_012026226.1	atgaactata	Rhamnogalacturonides degradation protein RhiN	17383	18603	+	872
6	WP_012026225.1	atgataaaga	Rhamnogalacturonides degradation protein RhiN	18616	19821	+	799
6	WP_044048002.1	atgtcggttta	Rhamnogalacturonides degradation protein RhiN	19871	21062	+	848

*Validated by qPCR
